# Fenofibrate Inhibited the Differentiation of T Helper 17 Cells *In Vitro*

**DOI:** 10.1155/2012/145654

**Published:** 2012-06-20

**Authors:** Zhou Zhou, Weiliang Sun, Ying Liang, Yanxiang Gao, Wei Kong, Youfei Guan, Juan Feng, Xian Wang

**Affiliations:** Department of Physiology and Pathophysiology, School of Basic Medical Sciences, Peking University and Key Laboratory of Molecular Cardiovascular Science, Ministry of Education, Beijing 100191, China

## Abstract

Uncontrolled activity of T cells mediates autoimmune and inflammatory diseases such as multiple sclerosis, inflammatory bowel diseases, rheumatoid arthritis, type 1 diabetes, and atherosclerosis. Recent findings suggest that enhanced activity of interleukin-17 (IL-17) producing T helper 17 cells (Th17 cells) plays an important role in autoimmune diseases and inflammatory diseases. Previous papers have revealed that a lipid-lowering synthetic ligand of peroxisome proliferator-activated receptor **α** (PPAR**α**), fenofibrate, alleviates both atherosclerosis and a few nonlipid-associated autoimmune diseases such as autoimmune colitis and multiple sclerosis. However, the link between fenofibrate and Th17 cells is lacking. In the present study, we hypothesized that fenofibrate inhibited the differentiation of Th17 cells. Our results showed that fenofibrate inhibited transforming growth factor-**β** (TGF-**β**) and IL-6-induced differentiation of Th17 cells *in vitro*. However, other PPAR**α** ligands such as WY14643, GW7647 and bezafibrate did not show any effect on Th17 differentiation, indicating that this effect of fenofibrate might be PPAR**α** independent. Furthermore, our data showed that fenofibrate reduced IL-21 production and STAT3 activation, a critical signal in the Th17 differentiation. Thus, by ameliorating the differentiation of Th17 cells, fenofibrate might be beneficial for autoimmunity and inflammatory diseases.

## 1. Introduction

Abnormal T-cell activity plays a central role in autoimmune diseases and inflammatory diseases [1]. Th17 cells represent a novel subset of CD4^+^ T cells, characterized by the secretion of a high level of IL-17. Despite controversial results [2], accumulating evidence has suggested that Th17 cells mediate various autoimmune and inflammatory diseases, including multiple sclerosis [3], inflammatory bowel diseases [4], rheumatoid arthritis [5], type 1 diabetes [6], and atherosclerosis [7–9], and interfering IL-17 may be beneficial for the above diseases.

 Atherosclerosis has long been recognized as a chronic autoimmune and inflammatory disease [10]. Although one pioneer study has shown a deteriorating effect by blocking Th17 cells [11], recent independent studies have indicated IL-17 as major pathogenic cytokine during atherogenesis [7–9]. Previously, we also demonstrated that hyperhomocysteinemia accelerates atherosclerosis development and vascular chronic inflammation by potentiating mitogen-induced proliferation and increasing IL-17 production of mouse T lymphocytes in ApoE^−/−^ mice [12, 13].

 Fenofibrate is a widely clinically used PPAR*α* agonist owing to its function of controlling hypertriglycemia [14]. By activating PPAR*α*, it also reduces inflammation because of suppressed NF-*κ*B activation [15–18]. Notably, this drug was also found to improve experimental autoimmune encephalomyelitis (EAE) [19] and autoimmune colitis [20], two autoimmune conditions in which Th17 cells play critical roles. Given precedent researches have shown that fenofibrate could probably influence the differentiation of the other two CD4^+^ effector T-cell groups, T helper 1 cells and T helper 2 cells [19, 21]. Our previous study has shown that fenofibrate enhances regulatory T-cell differentiation, and we hypothesize that fenofibrate may also possibly interfere with Th17 cell differentiation.

 Peripheral differentiation is the core step regulating the amount of Th17 cells, which is initiated by the stimulation of TGF-*β* and IL-6 upon T-cell activation [22, 23]. During this process, STAT3 activation plays a critical role [22, 23]. STAT3 is initially activated by IL-6, subsequently by autocrine IL-21, and the differentiation is finally determined by its master transcription factor ROR*γ*t [22, 23].

 In the present study, we found that fenofibrate markedly inhibited Th17 cell differentiation *in vitro.* This effect of fenofibrate might be attributed to the reduced activation of STAT3 and decreased production of IL-21. However, other PPAR*α* activators might not possess the same activity. These data indicate a new mechanism of fenofibrate to exert its anti-inflammatory effect.

## 2. Materials and Methods

### 2.1. Animals

Six-week-old female C57BL/6 mice of special pathogen-free level were purchased from the Animal Center of Peking University Health Science Center (Beijing, China). This study was carried out in strict accordance with the recommendations in the Guide for the Care and Use of Laboratory Animals of the Health Science Center of Peking University. The protocol was approved by the Committee on the Ethics of Animal Experiments of Peking University Health Science Center. All surgeries were performed with mice under anesthesia with sodium pentobarbital, and all efforts were made to minimize suffering.

### 2.2. Cell Sorting and Induction of Th17 Differentiation *In Vitro*

After the mice were sacrificed, the total and CD4^+^ splenic T cells were purified with positive selection microbeads against CD90.2 and CD4 (Miltenyi Biotec, Bergisch Gladbach, Germany), respectively, following the manufacturer's instructions. Then the cells were cultured as performed previously [24] with brief modifications. 1 × 10^6^ total T cells or CD4^+^ T cells were seeded with RPMI 1640 medium (Hyclone, Carlsbad, CA) containing 10% fetal bovine serum (Hyclone, Carlsbad, CA) in 48-well plates containing 1 *μ*g/mL plate-bound anti-CD3 (BD Pharmagen, Franklin Lakes) and 1 *μ*g/mL soluble anti-CD28 (BD Pharmagen, Franklin Lakes) antibodies. For Th17 differentiation, cultures were added with 10 ng/mL TGF-*β*1 (Pepro Tech, Rocky Hill) and 40 ng/mL IL-6 (Pepro Tech, Rocky Hill). 5 *μ*g/mL anti-interferon-*γ* (IFN-*γ*) antibody (R&D Systems, Minneapolis, MN) and 5 *μ*g/mL anti-IL-4 antibody (R&D Systems, Minneapolis, MN) were added as indicated. Fenofibrate, WY14643, GW7647, and bezafibrate (all from Sigma Chemical Co., St. Louis, MO) were used as the doses indicated at the beginning of the induction. For flow cytometry and RT-PCR, cells were cultured for 4 days. For western blot and ELISA, the culture lasted 1 or 2 days.

### 2.3. Flow Cytometry Analysis

For IL-17 staining, brefeldin A (Biolegend, San Diego, CA), 50 ng/mL PMA (Sigma Chemical Co., St. Louis, MO), and 1 *μ*g/mL ionomycin (Sigma Chemical Co., St. Louis, MO) were added. Then, 5 hours later, cells were collected and stained with Alexa Fluor 647-tagged anti-IL-17 antibody (BD Pharmagen, Franklin Lakes). For CD4 and CD8 staining, cells were first stained with FITC tagged CD4 antibody (eBioscience, San Diego, CA), PE-tagged CD8 antibody (eBioscience, San Diego, CA), and then Alexa Fluor 647-tagged anti-IL-17 antibody according to the manufacturer's instructions. For gp80 and gp130 staining, cells were collected 24 and 48 hours after the initiation of Th17 differentiation. PE tagged anti-gp80 and APC-tagged anti-gp130 antibody were used according to the instructions of the manufacturer.

### 2.4. Real-Time RT-PCR Analysis

 Cells were collected 4 days after the induction of Th17 cell differentiation, and total RNA was extracted with TRIzol reagent (Invitrogen, Carlsbad, CA) as previously described [25]. Then, AMV reverse transcription system (Promega, Madison, WI) was introduced to perform the reverse transcription of one microgram of RNA per sample. Real-time PCR amplifications involves an Mx3000 multiplex quantitative PCR system (Stratagene Corp, La Jolla, CA) and SYBR Green I reagent.

All amplification reactions carried out for 40 cycles were performed in duplicate (an initial stage of 7 min at 95°C, followed by a three-step cycle of 20 s at 94°C, 25 s at 60°C, and 30 s at 72°C). The accuracy of PCR products was confirmed by sequencing of the amplicons. The relative target mRNA levels normalized to that of the internal control *β*-actin were assessed with Stratagene Mx3000 software. The primers were used as follows: ROR*γ*t forward, AATGGAAGTCGTCCTAGTCAG, and reverse, CCGTGTAGAGGGCAATCTCA; *β*-actin forward, ATCTGGCACCACACCTTC, and reverse, AGCCAGGTCCAGACGCA.

### 2.5. ELISA Analysis

Supernatant of cell culture 2 days after the initiation of Th17 cell differentiation was collected, and ELISA was performed with mouse IL-21 quantifying kit (eBioscience, San Diego, CA) following the instructions of the manufacturer.

### 2.6. Western Blot Analysis

 Immunoblotting was performed as described previously [25]. Briefly, T-cell lysis samples containing the same amount of protein were resolved in 10% SDS-PAGE. The membranes were incubated with primary antibodies and then IRDye 700DX-conjugated secondary antibodies (Rockland Inc, Gilbertsville, PA). The immunofluorescence signal was detected by the Odyssey infrared imaging system (LICOR Biosciences, Lincoln, NB). The primary antibodies include anti-total, anti-phosphorylated STAT3 antibodies (Cell Signal Technology, Danvers, MA) and anti-eIF5 antibody (Santa Cruz, CA).

### 2.7. Transfection and Luciferase Reporter Assay

Transfection and luciferase reporter assays were performed as described [26] with brief modifications. Mouse embryonic fibroblast cells (MEF) were transfected with 0.2 *μ*g peroxisome proliferator response element (PPRE) luciferase reporter plasmid, together with *β*-galactosidase-expressing plasmid as an internal reference with cationic polymer transfection reagent (JetPEI, France). After transfection for 4 hours, the cells were incubated with fresh Dulbecco modified Eagle medium (DMEM) containing 10% fetal bovine serum and fenofibrate, WY14643, GW7647, or bezafibrate. 24 hours later, the cells were collected, and the luciferase activity relative to *β*-galactosidase activity was measured by luciferase assay system (Promega, Madison, WI).

### 2.8. Cell Viability and Proliferation Assays

Cells were collected 4 days after the Th17 differentiation. Annexin V/PI staining (Invitrogen, Carlsbad, CA) was used to verify the cell viability. The double-positive cells were taken as dead cells and the double-negative cells were taken as viable cells. For proliferation assays, CCK-8 staining was used according to the instructions of the manufacturer.

### 2.9. Statistical Analysis

 All data were expressed as mean ± SEM or original data representing one of at least three independent experiments. One-way ANOVA followed by Newman-Keul's post hoc test was used to compare multiple groups. Unpaired Student *t*-test was performed to compare two groups. *P* < 0.05 was considered statistically significant.

## 3. Results

### 3.1. Fenofibrate Inhibited the Differentiation of Th17 Cells *In Vitro*

**Figure 1:**
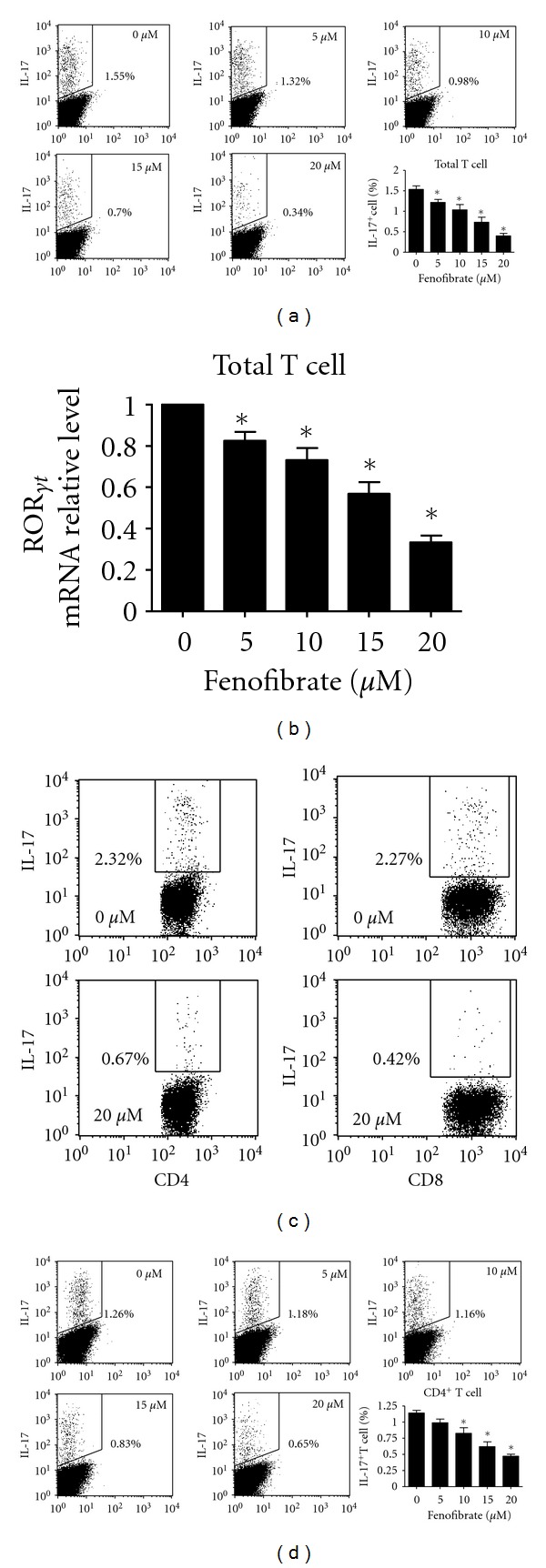
Fenofibrate inhibited the differentiation of Th17 cells* in vitro*. (a) Total T cells were isolated from mouse spleens and induced differentiation to IL-17^+^ cells with 10 ng/mL TGF-*β*, 40 ng/mL IL-6, and fenofibrate final concentrations indicated. The percentage of IL-17^+^ cells was analyzed with flow cytometry 4 days later. Total T cells were treated as above, the mRNA level of ROR*γ*t was analyzed with real-time PCR (b), and the percentages of IL-17^+^ cells in CD4^+^ and CD8^+^ T cell subgroups were analyzed separately according to different staining patterns with flow cytometry (c). (d) CD4^+^ T cells were isolated from mouse spleens and induced to differentiate into Th17 cells with 10 ng/mL TGF-*β*, 40 ng/mL IL-6, and fenofibrate final concentrations indicated. The percentage of Th17 cells was analyzed with flow cytometry 4 days later. In (a and d), numbers at the corner indicate the final concentration of fenofibrate, and the percentages of IL-17^+^ cells were shown adjacent to the outlined areas, *n* = 3 ~ 4, *P < 0.05 versus 0 *μ*M group. In (b), *n* = 3, *P < 0.05 versus 0 *μ*M group. In (c), data represent one of three independent experiments.

To determine the function of fenofibrate on the differentiation of Th17 cells, we first adopted an *in vitro* induction system supplemented with TGF-*β* and IL-6 [24]. The percentage of IL-17^+^ cells was analyzed with flow cytometry. Fenofibrate concentration dependently (5 ~ 20 *μ*M) reduced the IL-17^+^ cell percentage differentiated from total T cells, and it at 20 *μ*M markedly reduced the differentiation of IL-17^+^ T cells by 74% (fenofibrate 0 versus 20 *μ*M, 1.54 ± 0.08% versus 0.40 ± 0.06%, *P* < 0.05, Figure 1(a)). As well, the mRNA level of the Th17 transcription factor ROR*γ*t was also greatly reduced (Figure 1(b)), which reinforced the repression of Th17 cell differentiation by fenofibrate. Next, we analyzed the origin of fenofibrate-responsive differentiated IL-17^+^ T cells. Fenofibrate at 20 *μ*M reduced the ratio of either CD4^+^ or CD8^+^ T cells to a similar level (Figure 1(c)); thus, the suppressive effect of fenofibrate on IL-17^+^ T-cell differentiation was not selective for the two subpopulations. To further confirm that fenofibrate suppressed Th17 differentiation, we found that fenofibrate could also suppress the differentiation of Th17 cells from purified CD4^+^ T cells (fenofibrate 0 versus 20 *μ*M, 1.14 ± 0.04% versus 0.47 ± 0.03%, *P* < 0.05) (Figure 1(d)). Therefore, fenofibrate inhibited the differentiation of Th17 cells from both total T cells and CD4^+^ T cells *in vitro*.

### 3.2. Suppression of Th17 Differentiation by Fenofibrate Was Independent of Th1 or Th2 Modulation

**Figure 2:**
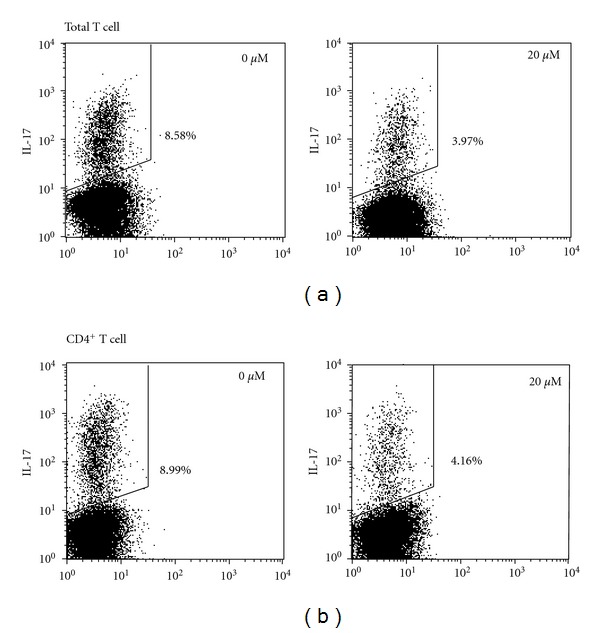
The effect of fenofibrate on Th17 cell differentiation did not involve changes of IFN-*γ* and IL-4 secretion. Total T cells (a) and CD4^+^ T cells (b) were isolated from mouse spleens and induced differentiation to Th17 cells with 10 ng/mL TGF-*β*, 40 ng/mL IL-6, 5 *μ*g/mL IFN-*γ* neutralizing antibody, 5 *μ*g/mL IL-4 neutralizing antibody, and fenofibrate final concentrations indicated. 4 days later, the percentage of IL-17^+^ T cells was analyzed with flow cytometry. Numbers adjacent to the outlined areas indicate the percentage of IL-17^+^ cells. Data shown here represent one of three independent experiments.

We next investigated the potential mechanisms by which fenofibrate inhibited Th17 differentiation *in vitro*. Previous papers have shown that fenofibrate modulated Th1 and Th2 cytokines, suppressing IFN-*γ* and supporting IL-4 expression, by activated T cells [19, 21]. IFN-*γ* and IL-4 have been suggested to inhibit the differentiation of Th17 cells [27, 28]. We therefore investigated whether fenofibrate regulated the differentiation of Th17 cells by modulating the production of these two cytokines. Application of neutralizing antibodies against IFN-*γ* and IL-4 exhibited no effect on Th17 cell differentiation from both total and CD4^+^ T cells (Figure 2), indicating that fenofibrate inhibited the differentiation of Th17 cells independent of the modulation of Th1 or Th2 cytokines.

### 3.3. WY14643, GW7647, and Bezafibrate Did Not Affect the Differentiation of Th17 Cells *In Vitro*

**Figure 3:**
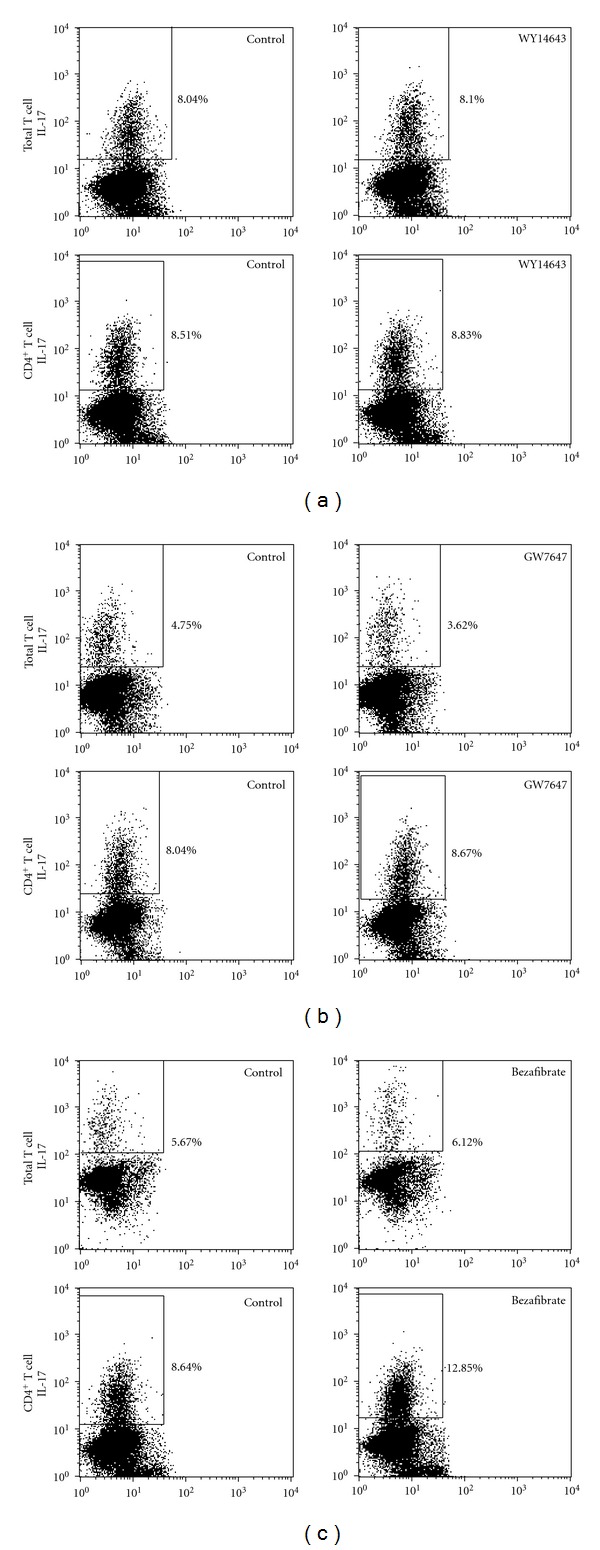
WY14643, GW7647, and bezafibrate did not inhibit the differentiation of Th17 cells. Total T cells and CD4^+^ T cells were isolated from mouse spleens and induced differentiation to Th17 cells with 10 ng/mL TGF-*β*, 40 ng/mL IL-6, 5 *μ*g/mL IFN-*γ* neutralizing antibody, 5 *μ*g/mL IL-4 neutralizing antibody, and 20 *μ*M WY14643 (a), 1 *μ*M GW7647 (b), 50 *μ*M bezafibrate (c) or solute control. 4 days later, the percentage of IL-17^+^ T cells was analyzed with flow cytometry. Numbers adjacent to the outlined areas indicate the percentage of IL-17^+^ cells. Data shown here represent one of three independent experiments.

Fenofibrate is classically defined as a PPAR*α* agonist [14]. To preliminarily decide the role that PPAR*α* played in the function of fenofibrate on Th17 differentiation, we tested the effect of other PPAR*α* activators in the differentiation system. Our data showed that none of WY14643 (20 *μ*M), GW7647 (1 *μ*M), or pan-PPAR agonist bezafibrate (50 *μ*M) downregulated the IL-17^+^ cell percentages (Figure 3). The efficacy of these PPAR agonists was reinforced by the upregulation of PPRE reporter luciferase activity (Supplemental Figure 1, see Figure S1 in Supplementary material available on line at doi:10.1155/2012/145654). Thus, PPAR*α* activation might not play a major role in the inhibitory effect of fenofibrate on Th17 differentiation. On the other hand, the data showed that none of the agonists influenced the viability or proliferation of T cells during Th17 differentiation (Supplemental Figures 2 and 3). 

### 3.4. Fenofibrate Reduced IL-21 Secretion in the Th17 Differentiation System

**Figure 4:**
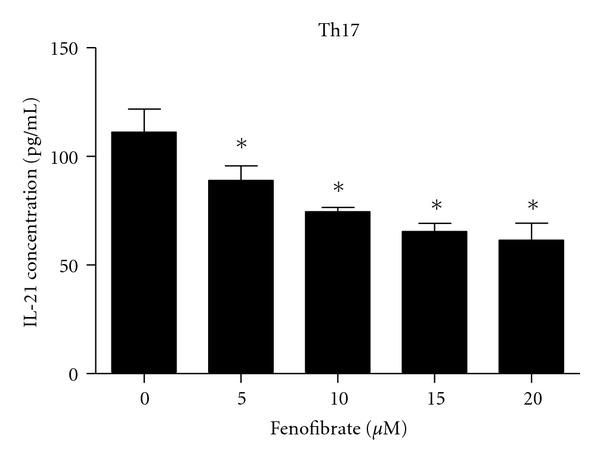
Fenofibrate reduced IL-21 production in the Th17 differentiation system. Total T cells were isolated from mouse spleens and induced differentiation to IL-17^+^ cells with 10 ng/mL TGF-*β*, 40 ng/mL IL-6, and fenofibrate final concentrations indicated. 2 days later, the concentration of IL-21 in the supernatant was examined with ELISA. *n* = 3, *P < 0.05 versus 0 *μ*M.

As a critical cytokine in the process of Th17 differentiation, IL-21 is autocrined by differentiating Th17 cells and ensures the differentiation of Th17 cells upon the activation by IL-6 and TGF-*β* [22]. Therefore, we next examined whether fenofibrate exerted its effect by influencing the production of IL-21. Our results showed that fenofibrate (5 ~ 20 *μ*M) concentration dependently decreased the IL-21 level in the Th17 differentiation system (Figure 4), which may explain, at least in part, the suppressive effect of fenofibrate on Th17 differentiation.

### 3.5. Fenofibrate Reduced the Activation of STAT3 during Th17 Differentiation

**Figure 5:**
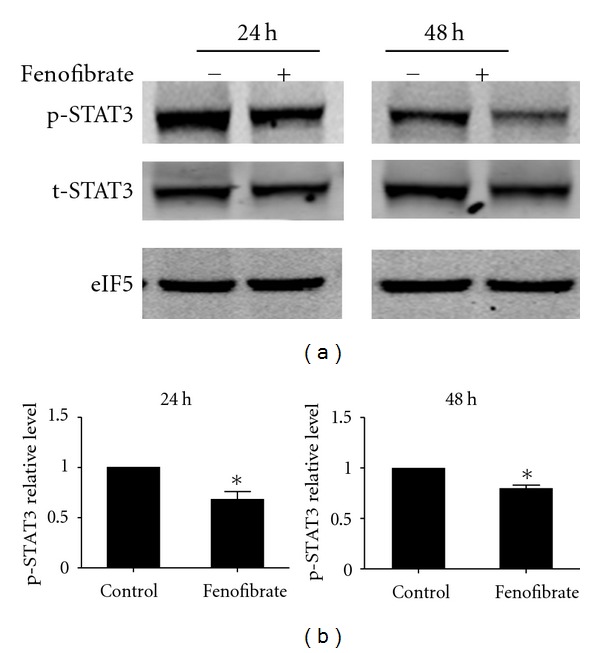
Fenofibrate reduced the activation of STAT3 in the Th17 differentiation system. (a) Total T cells were isolated from mouse spleens and induced differentiation to Th17 cells with 10 ng/mL TGF-*β* and 40 ng/mL IL-6 plus 20 *μ*M fenofibrate or solute control. 24 and 48 hours later, the phosphorylation state of STAT3 was analyzed with western blot. Data shown here represent one of three independent experiments. Graphs in (b) indicate the statistic significance of STAT3 phosphorylation state relative to the control group. *n* = 3-4, *P < 0.05 versus control.

Phosphorylation of STAT3, the downstream signal of IL-6 and IL-21, is a key process in the differentiation of Th17 cells in the presence of TGF-*β* [22, 23]. We therefore further examined whether fenofibrate influenced STAT3 activation. Our data showed that the phosphorylation levels of STAT3 were suppressed by 20 *μ*M fenofibrate at 24 and 48 hours, respectively (Figure 5), suggesting that fenofibrate may reduce the differentiation of Th17 cells through inhibiting STAT3 phosphorylation.

## 4. Discussion

Th17 cells play an essential role in the self-immune response and contribute directly to a variety of human autoimmune diseases and inflammatory diseases. In the present study, we have demonstrated that lipid-lowering drug fenofibrate inhibits the differentiation of Th17 cells *in vitro*. This effect of fenofibrate might be ascribed to reduced STAT3 activation and IL-21 secretion, but not the influence of IFN-*γ* and IL-4 secretion. PPAR*α* activation might play a minor role in its function because other PPAR*α* activators, WY14643 and GW7647, as well as pan-PPAR agonist bezafibrate did not exhibit the same function. These findings suggest a new mechanism by which fenofibrate exerts its anti-inflammatory effects *in vitro*.

Fenofibrate has been found to suppress inflammation and autoimmunity because of its potential to activate PPAR*α*. Activated PPAR*α* can inhibit the transcriptional activity of nuclear factor NF-*κ*B [15–18]. By activating PPAR*α*, fenofibrate also inhibited IFN-*γ*, the Th1 cytokine expression by activated cells [19, 21]. In addition, fenofibrate has also been found to exert some functions independent of PPAR*α* activation, including exacerbating left ventricular dilation and fibrosis in chronic pressure overload [29], inhibiting the production of cysteinyl leukotriene in mast cells [30], improving the survival of retinal endothelial cells [31], reducing the expression of plasminogen activator inhibitor type-I in progressive fibrosing steatohepatitis liver cells [32] and promoting the proliferation of liver cells [33]. In the present study, repressing Th17 cell differentiation might be another PPAR*α*-independent function of fenofibrate, despite the present finding that activating multiple nuclear receptors with their ligands ameliorate the differentiation of Th17 cells, such as RAR [34], RXR [34], AHR [35], LXR [36], and even two other members of PPAR family, PPAR*γ* and PPAR*β*/*δ* [37, 38].

The differentiation of Th17 cells depends critically on the signal transduction of STAT3. Activated STAT3 can directly induce the production of IL-17 and IL-21, and facilitate the expression of transcription factor ROR*γ*t, upon which the lineage of Th17 cells commits [22, 39]. Previous studies have revealed that several molecules can regulate the differentiation by interfering with the STAT3 signal. SOCS3, an endogenous repressor of STAT3, has been shown to inhibit the differentiation of Th17 cells because the deletion of SOCS3 led to enhanced STAT3 phosphorylation and then Th17 differentiation [11, 40]. Signal molecules like GSK3 [41] and SHIP [42] also support the differentiation of Th17 cells by ensuring the phosphorylation of STAT3. Several endogenous and exogenous noncytokine compounds such as retinoic acid [43], copolymer I [44], and glucuronoxylomannan [45] also repress Th17 differentiation through blocking STAT3 activation. In our present study, we have also observed suppressed phosphorylation of STAT3 during IL-6 induced Th17 differentiation, which might be the major pathway that fenofibrate exerted this very function. Although previous observations suggest that fenofibrate can reduce the activation of STAT3 in liver due to gp80 and gp130 suppression [46] depending on PPAR*α*, and that elevated activation of PPAR*α* in cardiomyocytes can suppress the activation of STAT3 by IL-6 [47], fenofibrate might suppress STAT3 activation through some other mechanisms because of the PPAR*α*-independent fashion. We have found that fenofibrate can suppress the secretion of the cytokine IL-21, another contributor of STAT3 signaling autocrined from differentiating Th17 cells [48], which might partially explain the function of fenofibrate. IL-6 is indeed the major source of the STAT3 signal; however, fenofibrate might probably suppress STAT3 activation via some other mechanisms. Collectively, these data put STAT3 as a central player in the inhibitory effect of fenofibrate on the differentiation of Th17 cells.

In addition, we tested the expression of SOCS3, an endogenous inhibitor of STAT3 activation, with western blot and found that SOCS3 protein level was not upregulated by fenofibrate (Supplemental Figure 4(a)). We also tested the membrane level of gp80 and gp130, as well as the phosphorylation of JAK1 and JAK2 (Supplemental Figures 4(b)–4(e)). None of them were affected by fenofibrate. The exact mechanism that fenofibrate inhibited STAT3 activation should be further studied. And whether and how fenofibrate interfere with autoimmune diseases in patients and animal models need further investigations too.

Apart from Th17 cells, some other T-cell subtypes also take part in inflammation and autoimmunity. Regulatory T-cell (Treg cell) is an important immune suppressive T-cell subtype. In our previous study, we have found that fenofibrate improves Treg* in vitro* differentiation via suppressing Akt and enhancing Smad3 activation [49]. Moreover, the *in vitro* differentiation of Th1 cell, another T helper subtype deteriorating inflammation and autoimmunity [50], can also be suppressed by fenofibrate (data not shown). These activities of fenofibrate are both also independent of PPAR*α*. Taken together, fenofibrate might be a special fibrate drug that not only lowers plasma triglyceride and reduces inflammation by activating PPAR*α* to improve cardiovascular diseases, but also regulates Th17, Th1 and Treg differentiation independent PPAR*α*.

Since the discovery of Th17 cells, strategies modulating the differentiation of them have been intensely investigated because of the anti-autoimmune and anti-inflammation potency. We have found that fenofibrate, a well-used hypolipidemic drug with minor adverse effects, suppresses Th17 differentiation *in vitro*. Thus, adding the anti-inflammatory effect via PPAR*α* discovered before by other groups, fenofibrate might also improve autoimmune and inflammatory diseases through multiple pathways including Th17 differentiation effects.

## Supplementary Material

The supplementary figures show the efficacy of PPAR activators, the viability and proliferation interference on T cells of the PPAR activators, and the expression patterns of SOCS3 and IL-6 receptor.
