# Supplementary material for: Fenofibrate Inhibited the Differentiation of T Helper 17 Cells In Vitro
Source: PPAR Res. 2012 Jun 20;2012:145654. doi: 10.1155/2012/145654 (PMC3388320; doi:10.1155/2012/145654)
Supplement: Supplementary file 1 — The supplementary figures show the efficacy of PPAR activators, the viability and proliferation interference on T cells of the PPAR activators, and the expression patterns of SOCS3 and IL-6 receptor. [file 145654.f1.pdf]

Supplemental Figure 1. Fenofibrate, bezafibrate, WY14643 and GW7647 activated PPARα .

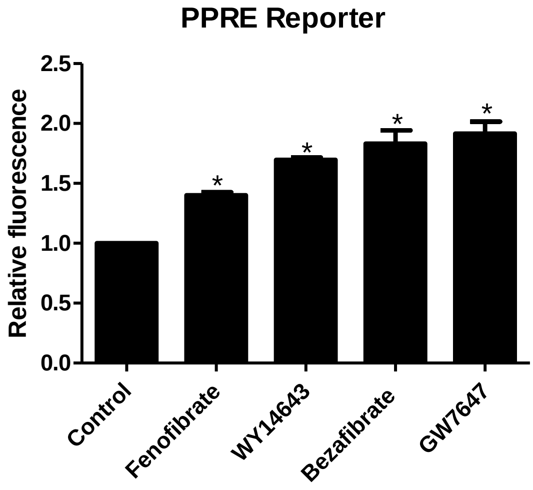

Supplemental Figure 2. Fenofibrate, WY14643, bezafibrate and GW7647 did not influence T cell viability.

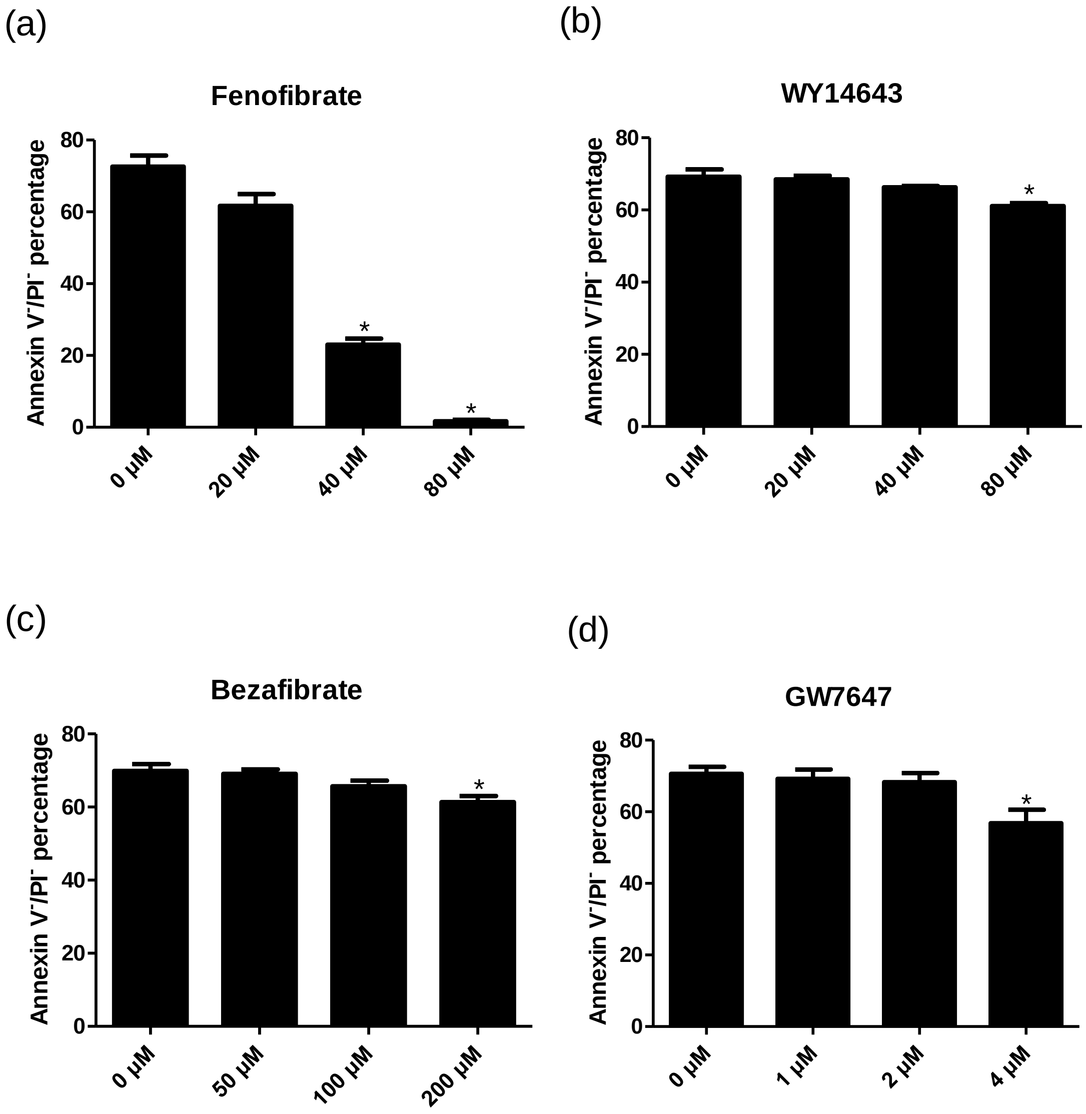

Supplemental Figure 3. Fenofibrate, WY14643, bezafibrate and GW7647 did not influence T cell proliferation.

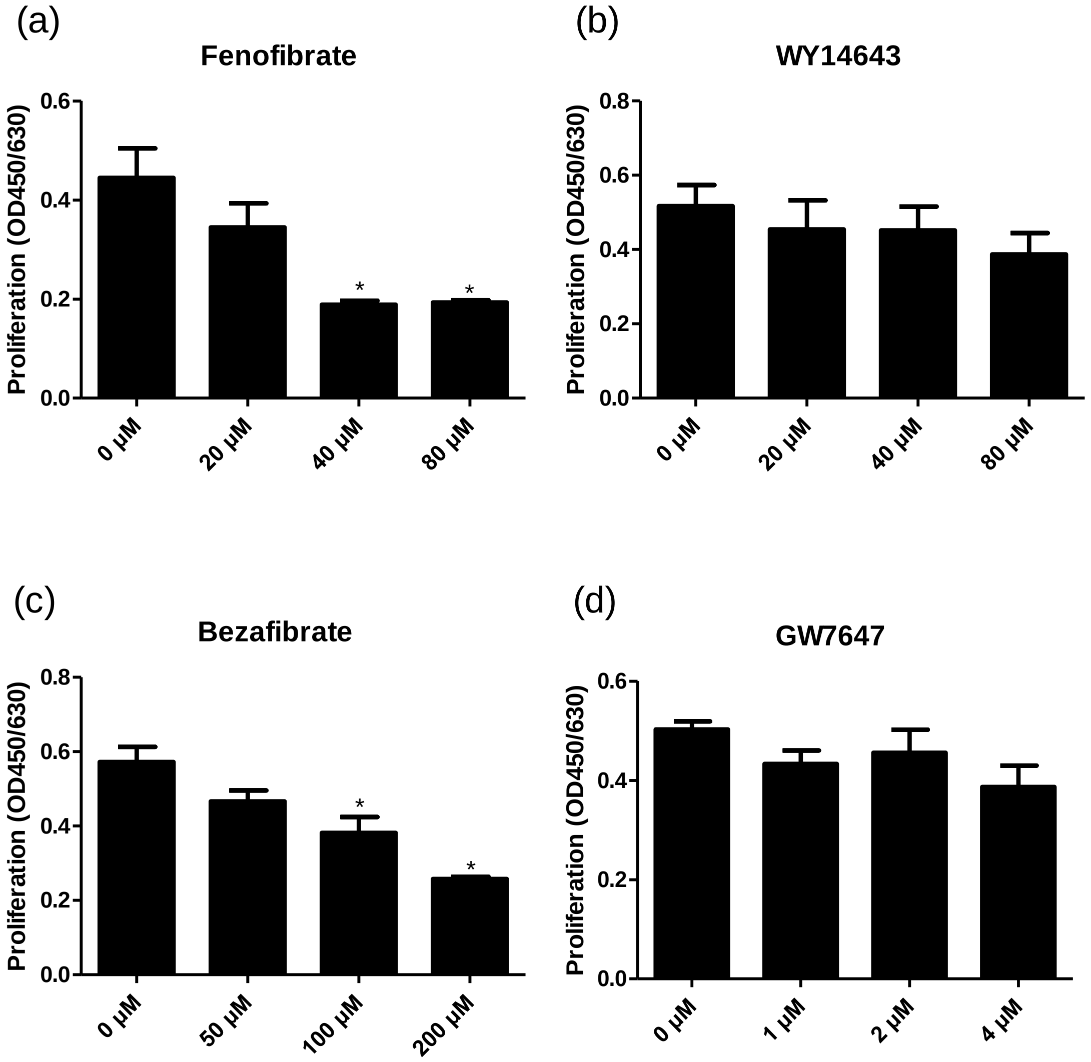

Supplemetal Figure 4. Fenofibrate did not affect the protein level of other members of IL-6 signal.

(a)

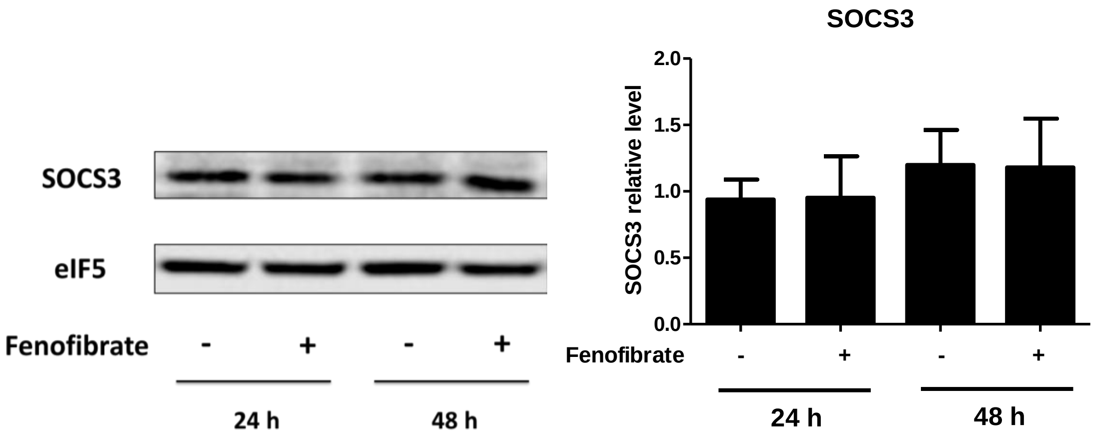

(b)

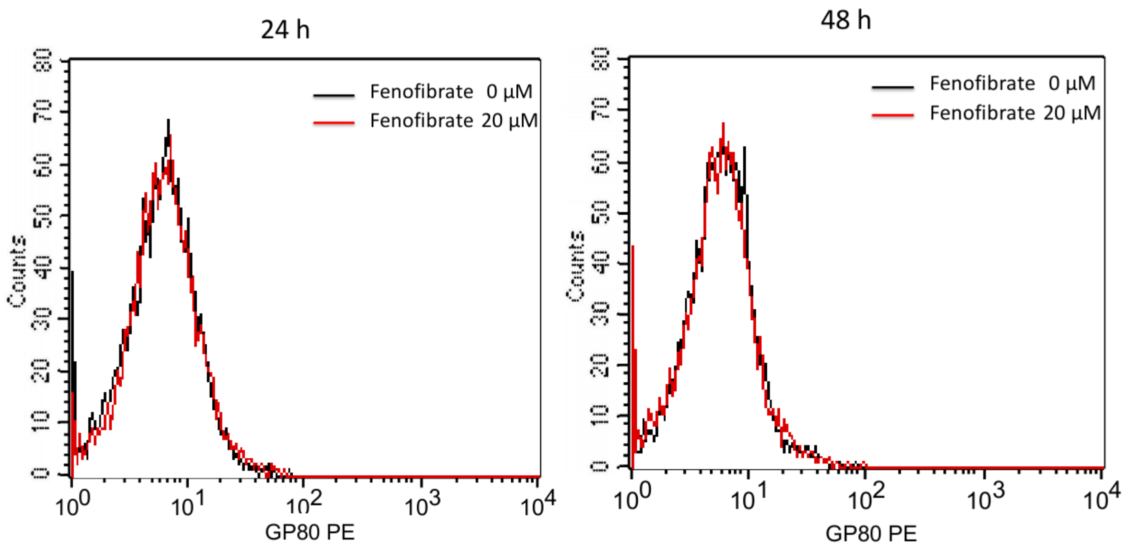

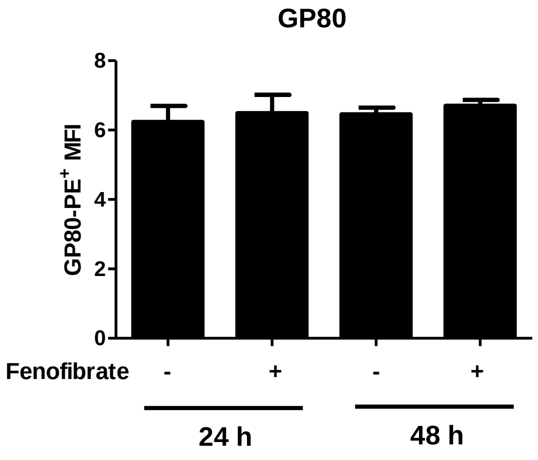
GP80
GP80-PE+ MFI
8
6
4
2
0
Fenofibrate
-
+
-
+
24 h
48 h

(c)

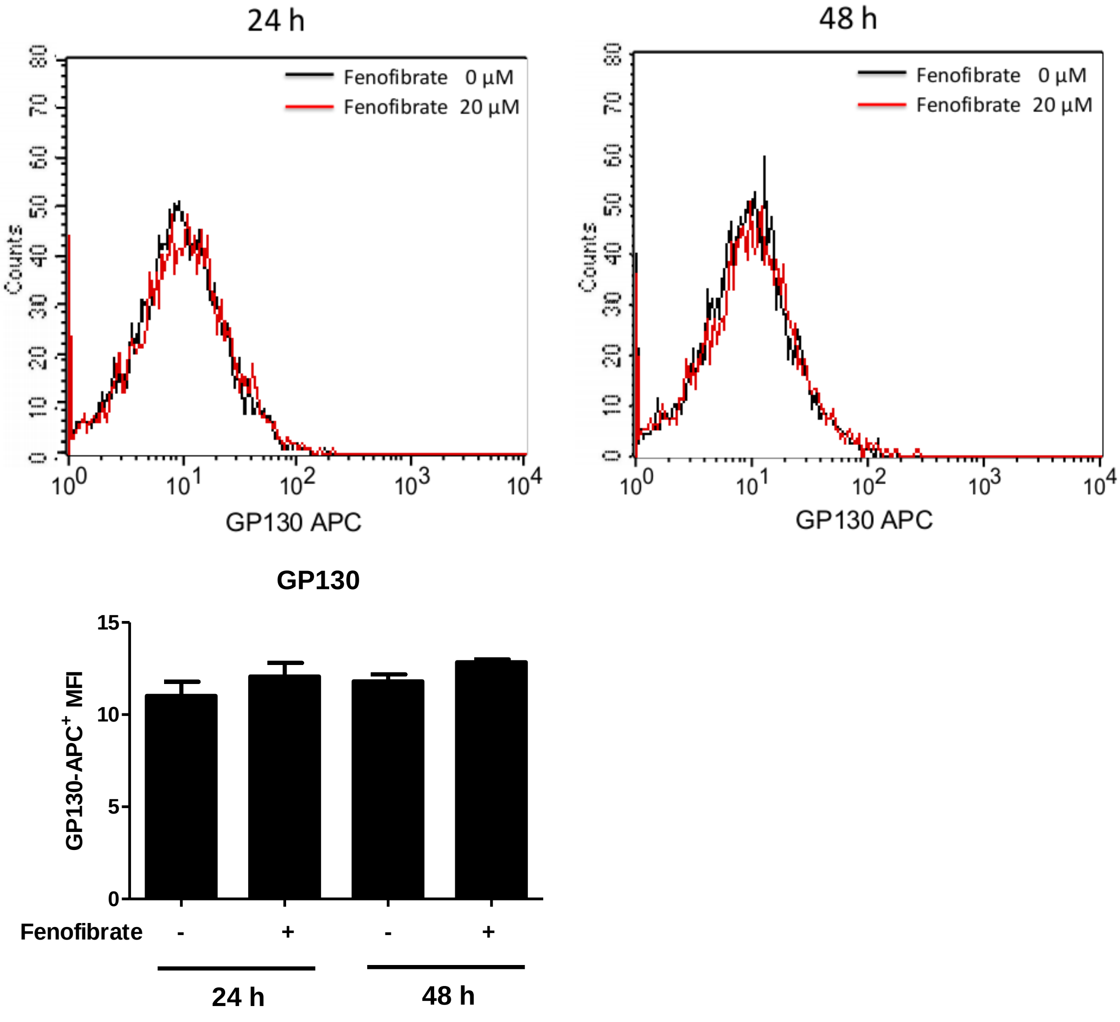
24 h
48 h
Fenofibrate 0 µM
Fenofibrate 20 µM
Counts
GP130 APC
GP130
GP130-APC+ MFI
15
10
5
0
Fenofibrate
-
+
-
+
24 h
48 h

(d)

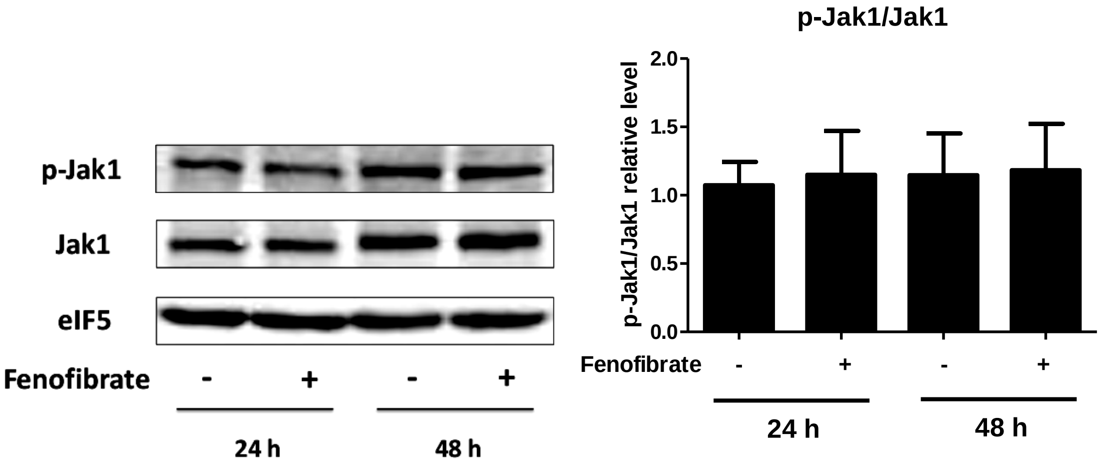
p-Jak1
Jak1
eIF5
Fenofibrate - + - +
24 h
48 h
p-Jak1/Jak1
p-Jak1/Jak1 relative level
2.0
1.5
1.0
0.5
0.0
Fenofibrate - + - +
24 h
48 h

(e)

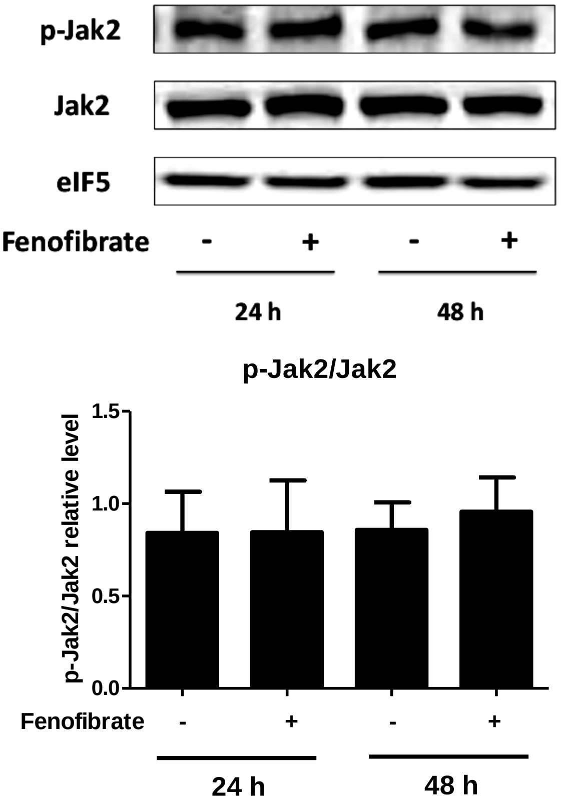
p-Jak2
Jak2
eIF5
Fenofibrate - + - +
24 h
48 h
p-Jak2/Jak2
p-Jak2/Jak2 relative level
1.5
1.0
0.5
0.0
Fenofibrate - + - +
24 h
48 h
